# Metabolomic Profiling of *Iris palaestina* via Molecular Networking and Its Anti-Diabetic Potential

**DOI:** 10.3390/molecules30122509

**Published:** 2025-06-08

**Authors:** Ayman Turk, Khodr Addam, Bang Yeon Hwang, Mi Kyeong Lee

**Affiliations:** 1 College of Pharmacy, Chungbuk National University, Cheongju 28160, Republic of Korea; aymanturk@chungbuk.ac.kr (A.T.); byhwang@chungbuk.ac.kr (B.Y.H.); 2 Faculty of Administrative Sciences, Global University, Beirut 15-5085, Lebanon; draddam@hotmaill.com

**Keywords:** *Iris palaestina*, molecular networking, phytochemical composition, α-glucosidase, metabolite

## Abstract

The *Iris* genus is known for its large blooms and significant conservation value, as well as its horticultural appeal. There are over 300 species of irises, which are widely distributed across the northern temperate zone. *Iris palaestina* (Baker) Barbey, commonly known as the Lebanese iris, is an endemic species of the Middle East with limited prior phytochemical research. This study was conducted to examine the metabolomic complexity and chemical profile of the flower extract of *I. palaestina* using advanced analytical tools. Molecular networking was employed to investigate its chemotaxonomy and phytochemical composition. In silico annotation tools—network annotation propagation (NAP), DEREPLICATOR, and MS2LDA—were applied to identify chemical classes and substructures within the extract. The flower extract of *I. palaestina* was found to contain diverse metabolite classes, including flavonoids, terpenoids, and lipids, with a total of 15 compounds annotated. Subsequent chromatographic separation yielded four major compounds, identified as the isoflavonoid irigenin, the flavonoid embinin, the xanthone mangiferin, and the lipid *N*-lauryldiethanolamine. Among these, irigenin and mangiferin exhibited significant α-glucosidase inhibitory activity, with IC_50_ values of 32.1 μM and 36.1 μM, respectively. This study provides the first comprehensive metabolomic characterization of *I. palaestina*, revealing it as a rich source of bioactive phytochemicals spanning multiple metabolite subclasses. These findings emphasize the possible use of *I. palaestina* for further pharmaceutical investigation and natural product discovery.

## 1. Introduction

The genus *Iris*, whose name originates from the ancient Greek term for rainbow, is a member of the Iridaceae family, consisting of blooming plants with conspicuous blossoms distinguished by a fragrance reminiscent of violets. Most *Iris* species prefer dry, rocky, or semiarid conditions; however, others do well in mesic or even wetland conditions [1]. The genus *Iris* comprises over 300 species distributed throughout temperate regions of the Northern Hemisphere, reflecting its remarkable diversity [2,3,4].

**Figure 1:**
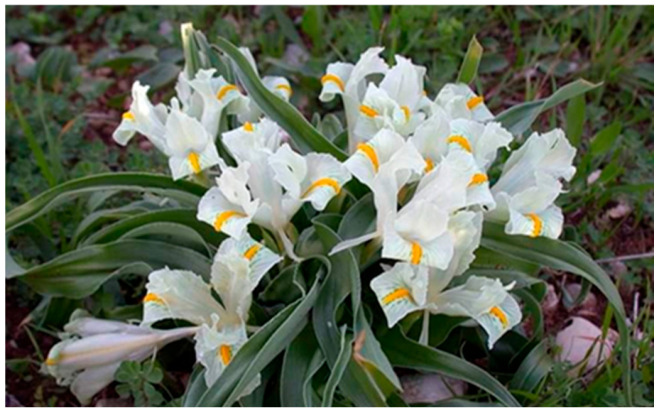
The upper parts of *I. palaestina* during the flowering period.

The Middle East region has many endemic species due to its geographical characteristics, but many plants have not been studied yet. In Lebanon, many plant species are well preserved, and it is estimated that there are 2600 species of vascular plants, of which 311 species (12%) are classified as endemic [5]. Currently, 389 species have been accepted in the *Iris* species, and a number of them are also found to be endemic to the Middle East region [1,6]. Fifty-three species in the genus *Iris* are now at risk of extinction on a global scale, with twenty-nine of them belonging to the section Oncocyclus [5]. Oncocyclus irises possess notable characteristics, such as their substantial and visually striking blooms, which have captivated horticulturists for an extended period [7]. Xerophytic plants are found in their native habitat in the Caucasus region, eastern Turkey, Syria, Lebanon, Jordan, and Palestine. From the genus *Iris*, around two hundred forty chemicals have been described; they include flavonoids, isoflavonoids and their glycosides, benzoquinones, benzene derivatives, triterpenoids, steroids, and stilbene glycosides [8,9,10]. *Iris* compounds have been shown to have anti-inflammatory, anti-plasmodial, antibiotic, and anti-tuberculosis effects [11,12]. *Iris palaestina* (Baker) Barbey [13] is a bulbous perennial species native to the Middle East, including Lebanon, and typically grows to a height of 10–20 cm from an ovoid bulb with light brown membranous tunics and fleshy storage roots. The flowers are characterized by small spreading or reflexed standards, recurved falls with blue-veined wings, and a distinctive, elevated yellow stripe that is purple-dotted but not crested; it is part of the rare and ecologically significant Oncocyclus irises (Figure 1).

Traditional herbal medicines have been thoroughly evaluated using metabolomics for their pharmacological efficacy and molecular processes in recent years. In addition, notable advancements in computational metabolome mining techniques have significantly influenced untargeted metabolomics data’s chemical and biological analysis. These techniques have effectively extracted functional information from spectrum data [14]. Because of their easy connection to annotation tools that provide automated mass spectral annotation and due to their capacity to offer a broad visualization of the substances within different biological systems, mass spectral mining strategies such as molecular networking and MS2LDA structure discoveries are gaining recognition in untargeted metabolomics [15,16]. Based on the idea that molecules with similar structures would have similar fragmentation patterns, molecular networking uses a vector-based computer technique to compare and assess the degree of spectrum similarity of a large mass spectrometry (MS) dataset [17,18]. Similar MS/MS spectra are clustered and shown in graph-based spectral networks so that the full spectrum of the mass spectrometer’s untargeted chemical fingerprints including known and unknown chemical repertoires may be seen [19].

*I. palaestina* is rare and has a small distribution; so, there are few studies on it. Therefore, in this study, we conducted research on the components and biological efficacy of this plant. In recent years, diabetes has emerged as a major global health concern due to its rising prevalence. α-Glucosidase enzymes play a key role in carbohydrate metabolism by catalyzing the breakdown of complex carbohydrates into absorbable monosaccharides. Therefore, inhibiting α-glucosidase activity represents an effective therapeutic strategy for managing postprandial hyperglycemia, as it slows glucose absorption in the intestine [20]. In this study, we evaluated the α-glucosidase inhibitory activity of metabolites isolated from *I. palaestina* to explore their potential as anti-diabetic agents. This study employed a molecular networking technique to annotate the metabolome of an *I. palaestina* flower extract. For their characterization, major components were purified using chromatographic techniques, and their structures were tentatively identified using spectroscopic analysis. The biological efficacy of the isolated compounds was also evaluated. Consequently, this study provides valuable information on the composition and biological efficacy of this particular species.

## 2. Results and Discussions

### 2.1. Metabolomic Profile of I. palaestina Flower Extract

**Figure 2:**
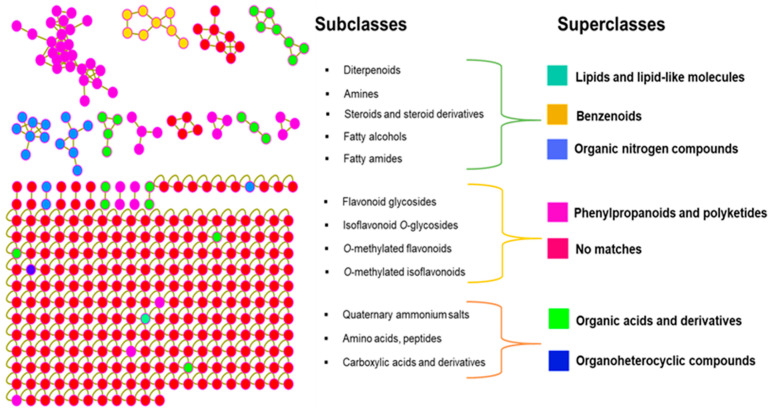
The network of the *I. palaestina* flower extract mass spectral features, represented by the MolNetEnhancer. Substructure annotations (MS2LDA), network annotation propagation (NAP), and DEREPLICATOR outputs were used to supplement the putative GNPS library’s match-based annotations of the nodes.

The methanol extract derived from the flowers of *Iris palaestina* (Figure 1) revealed a chemically rich and diverse metabolite profile. To comprehensively explore this metabolic landscape, the spectral data from the extract were analyzed using feature-based molecular networking within the GNPS platform. This approach allowed for a visual representation of the chemical space and facilitated the tentative identification of bioactive compounds. To enhance the reliability of the annotations observed in positive ion mode and to uncover additional metabolites, molecular networking was employed on the full dataset. A cosine similarity threshold of 0.5 was applied to ensure meaningful connections between spectral features. For clarity and precision, only clusters containing at least two interconnected features were considered in the analysis. The resulting molecular network (Figure 2) comprised 344 nodes, each representing a unique molecular feature. Among these, 15 nodes matched known metabolites based on spectral library comparisons and database annotations (Table 1), confirming the presence of several previously reported phytochemicals in *I. palaestina*. These findings demonstrate the utility of molecular networking in mapping the complex metabolome of underexplored plant species.

To further elucidate the chemical architecture of the *I. palaestina* flower extract, fragment-level analysis was performed using MS2LDA in combination with in silico annotation tools, including network annotation propagation (NAP) and DEREPLICATOR. These tools were integrated with feature-based molecular networking results through the MolNetEnhancer workflow, which facilitated the classification of compounds into chemical superclasses. By merging spectral library matches, predicted substructures, simulated fragmentations, and ontology-based chemical classifications [21], MolNetEnhancer enabled a comprehensive interpretation of the extract’s metabolomic landscape. This integrative approach revealed, for the first time, that the metabolome of *I. palaestina* is characterized by broad chemical diversity. The identified superclasses included lipids and lipid-like molecules, benzenoids, organic nitrogen compounds, phenylpropanoids and polyketides, organic acids, and organoheterocyclic compounds. Among these, phenylpropanoids and polyketides emerged as the most abundant chemical superclasses, followed by organic nitrogen compounds (Figure 2), a distribution pattern consistent with the metabolomic profiles previously reported for other *Iris* species [22]. At a more refined level, MolNetEnhancer also predicted subclass annotations, highlighting the presence of various flavonoid-related compounds such as flavonoid glycosides, *O*-methylated flavonoids, isoflavonoids, and derivatives of linoleic acid. These findings emphasize the chemical richness of *I. palaestina* and its potential as a source of bioactive metabolites with pharmaceutical relevance.

**Figure 3:**
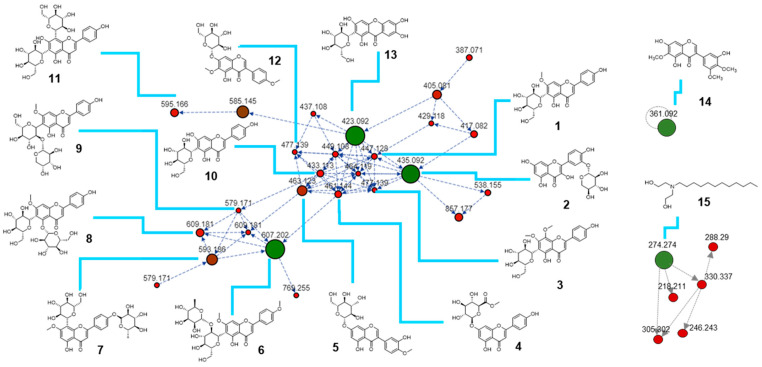
Molecular networking of the biggest cluster annotated as flavonoid and xanthone glycosides derived from the *I. palaestina* flower extract, obtained from the GNPS platform and visualized with Cytoscape 3.9.0 software.

Phenylpropanoids and polyketides represent a prominent class of natural products, with flavonoids being key constituents. As shown in Figure 3, the molecular network derived from the flower extract of *I. palaestina* revealed a prominent cluster distinguished by consistent fragmentation patterns and shared substructures. Tentative metabolite annotations were performed through comprehensive interpretation of the fragmentation behavior and by cross-referencing multiple mass spectrometry databases [23,24]. Mass spectrometry-based metabolomics enabled the detection and potential categorization of 15 metabolites in the flower parts, as shown in Table 1. Through mass spectrometry-based metabolomic analysis, a total of 15 metabolites were putatively identified in the flower parts (Table 1). These metabolites were grouped into three distinct clusters within the molecular network. The largest cluster consisted predominantly of glycosylated flavonoids (compounds **1**–**4** and **6**–**11**), isoflavonoids (**5** and **12**), and a single xanthone (**13**). Notably, quercetin 3′-xyloside (**2**), embinin (**6**), and mangiferin (**13**) were identified as key compounds within this dominant cluster, serving as central nodes due to their structural connectivity. Irigenin (**14**), a flavonoid aglycone, was observed as an isolated node outside the major clusters, while *N*-lauryldiethanolamine (**15**), a lipid, was identified as another key metabolite within a separate cluster. The observed clustering pattern appeared to correlate with core structural similarities and the presence or absence of sugar moieties. Specifically, the glycosylation pattern—whether *O*- or C-linked—as well as the number and position of methoxy or hydroxyl substituents contribute to the structural diversity of these metabolites. Flavonoids and isoflavonoids, while closely related, are differentiated by the position of the B-ring attachment on the central skeleton. Flavonoids are widely recognized for their antioxidant properties, while isoflavonoids function as phytoestrogens involved in bone metabolism and hormone regulation. Overall, the analysis confirmed that *I. palaestina* contains a chemically diverse array of flavonoid and isoflavonoid derivatives, many of which exist as glycosides. The structural variability observed—including sugar type, glycosylation position, and other substituents—contributes significantly to the phytochemical richness of this species and underscores its potential as a source of bioactive compounds.

### 2.2. Anti-Diabetic Potential of the Major Compounds

**Figure 4:**
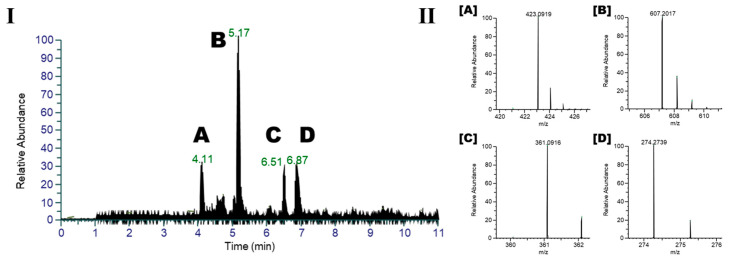
(**I**) HRESI-MS/MS chromatograms of *I. palaestina* flower extract, showing four major peaks corresponding to identified metabolites: (A) mangiferin, (B) embinin, (C) irigenin, and (D) *N*-lauryldiethanolamine. (**II**) MS/MS fragmentation spectra of compounds A–D, with [M + H]^+^ adduct ions and predicted molecular formulas annotated based on high-resolution accurate mass measurements and diagnostic fragmentation patterns.

In the present study, we investigated the biological efficacy of the major metabolites tentatively identified in *I. palaestina*, focusing on their anti-diabetic potential. Experimental and clinical investigations have validated the anti-diabetic properties of herbal extracts and chemicals derived from medicinal flora. Dietary flavonoids are recognized for their significance in preventing degenerative illnesses [25,26]. Moreover, epidemiological studies indicate that an increased intake of phenolic compounds correlates with a reduced prevalence of type 2 diabetes mellitus [22,27]. Therefore, we purified the major compounds and measured their inhibitory effects on α-glucosidase, which is an important target in diabetes therapy. Four major compounds that were identified by LC/MS analysis (Figure 4) were purified from the *I. palaestina* flower extract using chromatographic techniques. The isolated compounds were identified as mangiferin (A, **13**), a xanthone derivative, embinin (B, **6**), a flavonoid, irigenin (C, **14**), an isoflavonoid, and *N*-lauryldiethanolamine (D, **15**), a lipid, consistent with the molecular networking in Figure 3. Among the major compounds, mangiferin (A) and irigenin (C) showed strong α-glucosidase inhibitory activity, with IC_50_ values of 36.1 and 32.1 μM, respectively, whereas embinin showed weak activity (Table 2). In comparison, the total methanolic extract of *I. palaestina* demonstrated moderate α-glucosidase inhibition, with an IC_50_ value > 100 μM.

### 2.3. Chemotaxonomic Implications of the I. palaestina Flower Metabolite Profile

The genus *Iris* comprises blooming plants characterized by conspicuous blossoms and fragrance. These plants have been known to contain diverse metabolites, which contribute to their biological activity. *Iris* has been reported to comprise about 300 species and is distributed worldwide [1,6]. Due to the phytochemical diversity across the *Iris* genus, research on the components of different species and their varietal comparisons has been actively pursued. While *Iris* species generally share a similar array of phytochemicals, differences in the relative abundance and chemical profiles of their major constituents have been documented [27,28,29,30,31].

Research on *Iris* sp. has been mainly conducted on the roots and not much on the aerial parts. The components of *Iris* sp. include xanthone, isoflavonoid, flavonoid, and phenolic compounds [29,32]. For instance, irigenin and its derivatives—classified as isoflavonoids—have been consistently reported in species such as *Iris confusa*, *Iris germanica*, and *Iris tectorum*, are associated with antioxidant and anticancer properties, and exert α-amylase inhibitory effects [31,33]. Flavonoids like embinin and its glycosides have also been isolated from *Iris persica* and *Iris pseudopumila*, contributing to their traditional medicinal uses [34]. Mangiferin, a xanthone, though more commonly found in *Mangifera indica*, has also been detected in *Iris unguicularis*, highlighting xanthones as minor but notable constituents of some *Iris* species [35]. In our study, many flavonoid glycosides were characterized, and xanthones, isoflavonoids, and fatty acids were also observed as major components of the examined extract, showing a similar pattern to those of previous studies on *Iris* sp. Our current study also identified mangiferin (A, **13**), a xanthone derivative, embinin (B, **6**), a flavonoid, irigenin (C, **14**), an isoflavonoid, and *N*-lauryldiethanolamine (D, **15**) as major components in *I. palaestina*. Although these compounds have previously been detected in other *Iris* species [31,33], their presence as dominant constituents in *I. palaestina* had not been reported to date, to the best of our knowledge. Our present study also led to the proposed annotation of several previously undocumented metabolites of the genus *Iris*. Quercetin 3′-xyloside (**2**), swertisin 8-methyl ether (**3**), apigenin-7-*O*-glucuronide methyl ester (**4**), and vicenin 2 (**11**) were first reported in *Iris* sp. in this study. Conclusively, the metabolite profile of *I. palaestina* showed important chemotaxonomic differences from those of other *Iris* species.

## 3. Materials and Methods

### 3.1. Plant Material

The flowers of *I. palaestina* were collected at Jiyeh, a coastal town in the Al Shouf District, lat 33.8449732, long 35.5143853, alt 20 m, about 23 km from Beirut to the south, in March 2020. The precise GPS coordinates for the collection site are 33°84′49″ N, 44°51′43″ E. A voucher specimen with the identification code CBNU-2021-03-IP is stored in the Herbarium of the College of Pharmacy, Chungbuk National University, Republic of Korea.

### 3.2. Extraction and Isolation

To purify the major compounds, the air-dried flowers of *I. palaestina* (95 g) were powdered and extracted three times with 80% MeOH (3 L) at room temperature for 24 h. The combined methanolic extracts were filtered and concentrated under reduced pressure at 40 °C using a rotary evaporator to yield a crude extract (9.2 g). The dried extract was di-luted in 80% MeOH, concentrated to 1 mg/mL, and then subjected to semi-preparative HPLC and eluted with MeOH-H_2_O (50:50), yielding compounds A (2.3 mg), B (3.1 mg), C (1.7 mg), and D (0.6 mg).

### 3.3. Analysis of the Chemical Profile Using LC-HRMS/MS

In the LC-HRMS/MS investigation, a Vanquish UHPLC system and diode array detector were used with an Orbitrap Exploris 120 mass spectrometer (Thermo Fisher Scientific, Waltham, MA, USA). The flowers of *I. palaestina* were analyzed using a YMC-Triart C18 column (YMC Inc., Devens, MA, USA) (100 × 2.1 mm, 1.9 μm). A gradient system consisting of water with 0.1% formic acid and acetonitrile with 0.1% formic acid was used in a ratio of 90:10 to 0:100. The analysis was performed at a flow rate of 0.3 mL/min. The concentration of the extracts used was 0.5 mg/mL. The column oven was warmed to a temperature of 30 °C, and the injection volume of the samples was established as 5 μL. The resolution of the Orbitrap mass analyzer (Thermo Fisher Scientific, MA, USA) was set to 60,000 for the whole MS scan and 15,000 for the data-dependent MSn scan. Mass detection was conducted within the *m*/*z* range of 200–2000. The ion source parameters for HESI included a spray voltage of 3.5 kV, a vaporizer temperature of 275 °C, an ion transfer tube temperature of 320 °C, a sheath gas flow rate of 6.4 L/min, an auxiliary gas flow rate of 12 L/min, and a sweep gas flow rate of 2.2 L/min. The Orbitrap detector (Thermo Fisher Scientific, MA, USA) facilitated ion collisions at a normalized higher energy collision dissociation (HCD) energy level of 30%. The MS2 spectra of the four most intense ions were obtained by MS/MS fragmentation employing the data-dependent MSn mode. Additionally, a dynamic exclusion filter was used to inhibit the subsequent fragmentation of the ions for 2.5 s after the acquisition of the MS2 spectrum.

### 3.4. General Experimental Procedure

The NMR signals were analyzed using a Bruker DRX 400 MHz spectrometer (Bruker-Biospin, Karlsruhe, Germany), with methanol-d4 serving as the solvent. The UV and IR spectra were acquired using the Jasco UV-550 (manufactured by JASCO, Tokyo, Japan) and Perkin–Elmer model LE599 (manufactured by Perkin–Elmer, Waltham, MA, USA) spectrometers. The LCQ Fleet and maXis 4G mass spectrometers, manufactured by Bruker Daltonics in Bremen, Germany, were used to acquire ESIMS and HRESI-TOF-MS data. Semi-preparative high-performance liquid chromatography (HPLC) was conducted using a Waters 515 HPLC pump (Waters Corp., Milford, MA, USA). equipped with a 996 photodiode array detector and controlled by Waters Empower software (Version 3.8.0). Chromatographic separation was achieved using a Gemini-NX ODS column with dimensions of 150 × 10.0 mm and 150 × 21.2 mm. The experiment included using aluminum plates that were pre-coated with Kieselgel 60 F254 (0.25 mm, Merck, Darmstadt, Germany) to conduct thin-layer chromatography (TLC).

### 3.5. Molecular Networking

The MS1 and MS/MS data were converted into the mgf format using MZmine (version 2.4.2) [36], followed by data alignment. Molecular networking (MN) was constructed via the GNPS platform with parent ion and MS/MS fragment ion tolerances set at 0.02 Da. The network generation parameters included a minimum cosine similarity score of 0.5, requiring at least four matching fragment ions and peaks. To enhance the spectral quality, the MS/MS data were filtered by retaining only the top six most intense peaks within a ±50 Da window of each spectrum. The resulting spectra were then compared against GNPS spectral libraries for annotation. The final molecular network was visualized using Cytoscape version 3.9.0

### 3.6. Measurement of α-Glucosidase Activity

The inhibitory activity of the samples against α-glucosidase was evaluated using the method described in [37], with slight modifications. α-Glucosidase derived from *Saccharomyces cerevisiae* (EC 3.2.1.20) was used. The assay was performed in a 96-well microplate format. Briefly, 10 µL of the test sample (dissolved in DMSO), 80 µL of phosphate buffer (100 mM, pH 6.8), and 10 µL of an α-glucosidase enzyme solution (1 U/mL in phosphate buffer) were mixed and preincubated at 37 °C for 15 min. The enzymatic reaction was initiated by adding 10 µL of a 10 mM *p*-nitrophenyl α-_D_-glucopyranoside (pNPG) solution as the substrate. After incubation at 37 °C for 20 min, the samples were measured at 450 nm using a microplate reader. Acarbose was used as a positive control. All assays were performed in triplicate.

### 3.7. Statistical Analysis

Statistics are presented using means and standard deviations. Substantial variations between means were assessed using Duncan’s multiple range tests at a significance threshold of *p* < 0.05. All experimental data are expressed as mean ± standard deviation (SD) of three independent replicates (n = 3).

## 4. Conclusions

The use of computational techniques to analyze the metabolome of *I. palaestina* facilitated the semi-automated tentative identification of known metabolites using spectrum library matching, while insight-derived molecular networks helped tentatively identify new structurally related compounds. The use of molecular networking with various computational methods within the GNPS platform holds significant promise for elucidating intricate plant metabolomes, thereby addressing knowledge deficiencies in both uncharted and partially examined metabolomes. This led to the proposed annotation of several previously undocumented metabolites in the genus *Iris*. The metabolite profile of *I. palaestina* showed important chemotaxonomic differences from those of other *Iris* species. Profiling the metabolites of *I. palaestina* suggested the presence of diverse skeletons of compounds such as flavonoids, isoflavonoids, xanthones, and lipids. The purification and analysis of the major compounds suggested their anti-diabetic potential, as indicated by their α-glucosidase inhibitory effects. Therefore, the present results provide important information about *I. palaestina* and suggest further exploration of *I. palaestina* and its metabolites for pharmaceutical applications.

## Figures and Tables

**Table 1:** Annotated compounds in the molecular networking of the examined *I. palaestina* flower extract.

Compound Number	[M + H]^+^ *m*/*z*	Molecular Formulas [M]	Adduct Types	Cosine Score	Compound Name
**1**	447.128	C_22_H_22_O_10_	[M + H]^+^	0.95	Swertisin
**2**	435.092	C_20_H_18_O_11_	[M + H]^+^	0.71	Quercetin 3′-xyloside
**3**	477.139	C_23_H_24_O_11_	[M + H]^+^	0.83	Swertisin 8-methyl ether
**4**	461.144	C_22_H_20_O_11_	[M + H]^+^	0.87	Apigenin-7-*O*-glucuronide methyl ester
**5**	463.123	C_22_H_22_O_11_	[M + H]^+^	0.92	Pratensein-7-*O*-glucoside
**6**	607.202	C_29_H_34_O_14_	[M + H]^+^	0.80	Embinin
**7**	593.186	C_28_H_32_O_14_	[M + H]^+^	0.94	Isoswertisin 4′-*O*-rhamnoside
**8**	609.181	C_28_H_32_O_15_	[M + H]^+^	0.86	Swertisin 5-*O*-glucoside
**9**	579.171	C_28_H_32_O_15_	[M + H]^+^	0.93	Swertisin 2″-*O*-arabinoside
**10**	433.113	C_22_H_22_O_10_	[M + H]^+^	0.92	Isovitexin
**11**	595.166	C_27_H_30_O_15_	[M + H]^+^	0.94	Vicenin 2
**12**	477.139	C_23_H_24_O_11_	[M + H]^+^	0.83	Irisolidone 7-*O*-glucoside
**13**	423.092	C_19_H_18_O_11_	[M + H]^+^	0.93	Mangiferin
**14**	361.092	C_18_H_16_O_8_	[M + H]^+^	0.78	Irigenin
**15**	274.271	C_16_H_35_NO_2_	[M + H]^+^	0.89	*N*-lauryldiethanolamine

**Table 2:** Inhibitory effect of major compounds on α-glucosidase activity. The values in the second column represent the mean inhibitory activity at 100 μM concentration (n = 3, mean ± SD).

Compounds	α-Glucosidase Inhibitory Activity (100 μM)	IC_50_ (μM)
Mangiferin (**A**)	83.9 ± 3.4	36.1 ± 2.8
Embinin (**B**)	28.2 ± 6.3	>100
Irigenin (**C**)	87.7 ± 6.6	32.1 ± 3.1
*N*-lauryldiethanolamine (**D**)	NT ^a^	NT ^a^
Acarbose ^b^	74.6 ± 3.4	65.8 ± 2.3

^a^ NT: not tested; ^b^ acarbose was used as a positive control.

## Data Availability

The data will be made available on request.
